# Association of renal hyperfiltration with incident proteinuria - A nationwide registry study

**DOI:** 10.1371/journal.pone.0195784

**Published:** 2018-04-13

**Authors:** Seung Min Lee, Ju-Young Park, Min-Su Park, Jong Heon Park, Minseon Park, Hyung-Jin Yoon

**Affiliations:** 1 Department of Biomedical Engineering, Seoul National University College of Medicine, Seoul, Republic of Korea; 2 Department of Biomedical Systems Informatics, Yonsei University College of Medicine, Seoul, Republic of Korea; 3 Big Data Steering Department, National Health Insurance Service, Wonju, Gangwon, Republic of Korea; 4 Department of Family Medicine, Seoul National University Hospital, Seoul, Republic of Korea; Istituto Di Ricerche Farmacologiche Mario Negri, ITALY

## Abstract

To elucidate the association between renal hyperfiltration (RHF) and incident proteinuria, the data from 11,559,520 Korean adults who had undergone health screenings ≥ 3 times between 2009 and 2014 and had glomerular filtration rate (GFR) ≥60 mL/min/1.73m^2^ and negative dipstick test for proteinuria at baseline, were retrospectively analyzed. GFR was estimated with the Chronic Kidney Disease Epidemiology Collaboration equation based on serum creatinine, and RHF was defined as GFR >95^th^ percentile adjusted for sex, age, body size, and diabetes and/or hypertension medication. The adjusted hazard ratio (aHR) of incident proteinuria in the RHF was 1.083 (95% CI, 1.069~1.097) compared to that of the non-RHF with Cox regression model. The association between RHF and incident proteinuria was not only in diabetic but also in non-diabetic subjects. This association was not observed in women (p for interaction <0.001). A reverse J-shaped association was found between the adjusted GFR slope and aHR of incident proteinuria. Both lower and higher GFR were associated with incident proteinuria in men. In conclusion, RHF was associated with incident proteinuria in men. Clinical studies are necessary to study whether the alleviation of RHF can prevent incident proteinuria.

## Introduction

Renal hyperfiltration (RHF) has long been considered as one of the mechanisms of the progression of chronic renal diseases after a certain degree of renal injury has developed [1]. RHF has been associated with various clinical conditions including pre-diabetes and diabetes, pre-hypertension and hypertension, and metabolic syndrome [2–6], as well as lifestyle factors like obesity, smoking, lack of physical activity, lower cardiopulmonary fitness, and increased dietary acid load mainly due to a Western-style diet [7–11]. The clinical implications of RHF are not yet clear in conditions other than diabetes, in which RHF is considered as one of the initiating mechanisms of renal complications [12]. Several cohort studies and meta-analyses have reported a reverse J-shaped association between estimated glomerular filtration rate (eGFR) based on serum creatinine level and mortality [13–19]. Although this association has been explained with the overestimation of true GFR by eGFR based on serum creatinine level in high-risk subjects with decreased muscle mass [15], RHF defined by skeletal muscle mass-adjusted criteria has been associated with higher long-term all-cause and cardiovascular mortality in a relatively healthy population [20]. However, the renal implications of RHF associated with lifestyle factors or clinical conditions other than diabetes have not been studied yet.

Additionally, chronic kidney disease (CKD), the precursor disease of end-stage renal failure which causes a very heavy socio-economic burden in most countries [21], is an emerging health-related issue because of its rapidly increasing incidence and prevalence worldwide and its association with cardiovascular morbidity and mortality [22, 23]. The identification and intensive control of CKD in the reversible stages are very important due to the irreversible nature of progressive CKD and its association with cardiovascular outcomes. Because it is well known that RHF precedes the development of albuminuria and renal dysfunction in diabetic CKD [24], RHF may be a marker of the early reversible stage of CKD caused not only by diabetes but also by other conditions. Proteinuria, which is one of main diagnostic criteria of CKD, is well-known risk factor for cardiovascular morbidity and mortality independent of CKD. Testing the possibility of RHF as an indicator of future development of proteinuria in early reversible stage has paramount importance for developing strategies to prevent proteinuria and its complications. Longitudinal studies on the association between incident proteinuria and RHF associated with conditions other than diabetes have not been reported.

To test the hypothesis that RHF predicts incident proteinuria in the general population, we analyzed the national health screening data of more than eleven million Korean adults with eGFR 60 mL/min/1.73 m^2^ or above and negative dipstick test for proteinuria at baseline, who had undergone health screenings three or more times at least six months apart between 2009 and 2014.

## Methods

In Korea, regular health screening at designated screening hospitals across the country is obligatory for: 1) the employed and the self-employed who are the householders of a family, biannually (annually for manual workers); 2) dependents of the employed and family members of the self-employed householder aged 40 years or older, biannually; 3) medical aid beneficiaries, biannually (householders 19~64 years of age and family members 41~64 years of age, since 2012)[25]. The National Health Insurance Service of Korea collects the health screening data from all the screening hospitals in Korea. Serum creatinine testing was first included in the national health screenings in 2009. Therefore, for this study, health screening data from the period between Jan 2009 and Dec 2014 were retrospectively analyzed. The number of eligible subjects between 2009 and 2014 was between 15,036,607 and 16,456,214, and the participation rate was between 66.0% and 74.8% [25]. The data of lifestyle, past medical history, current medical conditions, such as presence or absence of major illnesses, and family history were collected using a structured questionnaire completed by the participants. Height, weight, waist circumference, blood pressure, visual acuity, and hearing ability were measured. Laboratory tests included fasting blood sugar, liver function test, blood hemoglobin, blood lipids, serum creatinine, urine protein by urine dipstick, and chest x-ray.

Between 2009 and 2014, 71,616,203 health screenings were performed in 27,448,308 out of approximately 50 million Koreans, of which approximately 36 million were 20 years of age or older. Health screening tests performed for foreign nationals and subjects younger than 20 years of age (1,404,421 screenings) and those with incomplete data (1,546,300 screenings; missing values in the adjusting variables included in statistical analyses) were excluded. The data of the 1,671,630 subjects with baseline eGFR lower than 60 mL/min per 1.73 m^2^ and/or baseline dipstick test for proteinuria trace or higher were excluded. The data from 11,559,520 subjects who had undergone health screenings three or more times at least six months apart between 2009 and 2014 were analyzed. The number of the subjects who had undergone health screening five or more times during this period was 2,793,037.

eGFR was calculated with the Chronic Kidney Disease Epidemiology Collaboration equation based on the serum creatinine level [26]. Serum creatinine measurements during the study period were mostly not standardized to isotope dilution mass spectrometry in Korea and were adjusted as previously described [19]. Proteinuria was defined as spot urine dipstick test for protein 1+ or higher and incident proteinuria as conversion of spot urine dipstick test, negative at the individual’s first health screening to 1+ or higher at any time during the follow-up period. RHF was defined as previously proposed with some modifications [11, 27] by using initial health screening data of each participant. Briefly, the residuals were calculated from a multiple linear regression analysis, in which the logarithm-transformed eGFR was a dependent variable and sex, weight, height, known history of medication for diabetes and/or hypertension, and logarithm-transformed age were independent variables. An eGFR with residuals higher than the 95^th^ percentile was defined as RHF. Participants without RHF were defined as non-RHF.

The association between RHF and incident proteinuria was analyzed with Cox regression model, adjusting for possible confounding variables at baseline, such as age, sex, body mass index (BMI), known history of medication for diabetes and/or hypertension, smoking status, regular alcohol consumption, regular exercise, systolic blood pressure, fasting serum glucose, serum triglycerides, and serum high-density lipoprotein-cholesterol. The relationship between the percentile rank of eGFR residual and the adjusted hazard ratio (aHR) of incident proteinuria was visualized with a generalized additive model, in which the possible confounding variables at baseline, such as age, sex, BMI, known history of medication for diabetes and/or hypertension, smoking status, regular alcohol consumption, regular exercise, systolic blood pressure, fasting serum glucose, serum triglycerides, and serum high-density lipoprotein-cholesterol were adjusted. The relationship between the eGFR slope and the aHR of incident proteinuria was visualized with the same method, except that baseline proteinuria was not included in the adjusting variables. In the Cox regression models, penalized splines as the smoothing were implemented by the R function pspline in package *survival* (degree of freedom was 6). For subgroup analyses, the total subjects were divided according to sex, sex-specific median age (44 years in men, 49 years in women), and the diabetic status (fasting serum glucose 126 mg/dL or above and/or anti-diabetic medication). For sensitivity analyses, the data of 2,793,037 subjects who had undergone health screening five or more times were analyzed for the association between RHF and proteinuria was defined as urine dipstick test for proteinuria 1+ or higher in two consecutive tests for the association between RHF and incident proteinuria. The association between RHF and incident proteinuria defined as urine dipstick test for proteinuria 2+ or higher was analyzed also. All statistical analyses were conducted using R 3.2.3 (http://www.R-project.org).

The Institutional Review Board of Seoul National University Hospital waived the informed consents and approval because of the nature of this study, which retrospectively analyzed the national registry data.

## Results

Table 1 shows the baseline characteristics of the subjects. The proportion of current smokers, those who drank more than three times per week, those who did not exercise regularly, and those taking anti-hypertensive or anti-diabetic medication was higher in the RHF group than in the non-RHF group. Systolic and diastolic blood pressure, BMI, and fasting serum glucose were higher in the RHF group than in the non-RHF group. The lipid profile was better in non-RHF than in RHF group (Table 1).

**Table 1:** General characteristics of the subjects at the initial health examination.

		Non-RHF	RHF^a^^)^	P-value^b^^)^
		(10,981,545)	(577,975)	
Sex (men)		5,996,470 (54.6%)	336,239 (58.2%)	0.992
Age (yr)		46.5 ± 13.4	46.5 ± 11.6	0.083
Smoking	Never	6,625,030 (60.3%)	327,063 (56.6%)	<0.001
	Former	1,627,609 (14.8%)	87,794 (15.2%)	
	Current	2,728,906 (24.8%)	163,118 (28.2%)	
Alcohol consumption (times/week)	None	5,574,269 (50.8%)	277,071 (47.9%)	<0.001
	1~2	3,991,594 (36.3%)	203,736 (35.2%)	
	3~4	1,033,707 (9.4%)	67,101 (11.6%)	
	>4	381,975 (3.5%)	30,067 (5.2%)	
Regular exercise^c^^)^		2,630,588 (24%)	132,642 (22.9%)	<0.001
Anti-hypertensive medication		1,391,621 (12.7%)	92,035 (15.9%)	<0.001
Anti-diabetic medication		448,592 (4.1%)	28,254 (4.9%)	<0.001
Height (cm)		164.1 ± 9.2	164.6 ± 9.1	<0.001
Weight (kg)		64.1 ± 11.5	65.5 ± 12.7	<0.001
Systolic blood pressure (mmHg)		121.9 ± 14.6	123.4 ± 15.0	<0.001
Diastolic blood pressure (mmHg)		76.1 ± 9.9	76.9 ± 10.3	<0.001
Body mass index (kg/m^2^)		23.7 ± 3.1	24.1 ± 3.5	<0.001
Fasting serum glucose (mg/dL)		96.4 ± 21.4	98.0 ± 25.0	<0.001
Serum triglycerides (mg/dL)		131.5 ± 90.7	137.3 ± 99.1	<0.001
Serum HDL-cholesterol (mg/dL)^d^^)^		55.1 ± 13.5	54.6 ± 14.2	<0.001
Serum creatinine (mg/dL)		0.87 ± 0.17	0.56 ± 0.11	<0.001
eGFR (mL/min/1.73 m^2^)^e^^)^		94.0 ± 14.7	118.7 ± 10.5	<0.001

^a^ Renal hyperfiltration, see Methods for details.

^b^ by t-test for continuous variables and chi-square test for categorical variables.

^c^ regular exercise: moderate intensity exercise or higher.

^d^ serum high-density lipoprotein-cholesterol.

^e^ estimated glomerular filtration rate by the Chronic Kidney Disease Epidemiology Collaboration equation based on serum creatinine.

**Fig 1:**
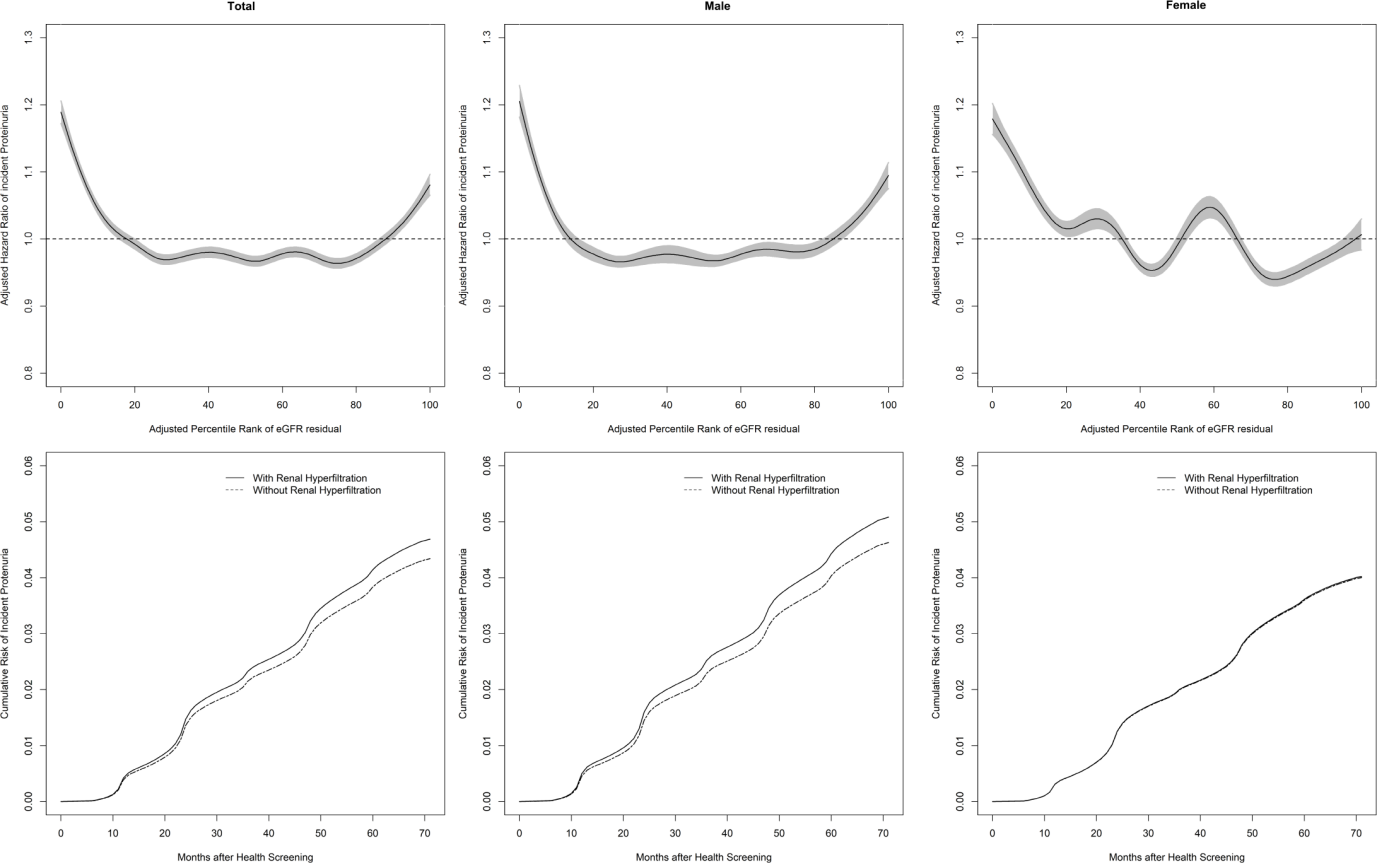
Association between renal hyperfiltration (RHF) with incident proteinuria. Upper panel. A reverse J-shaped association between baseline eGFR and the adjusted hazard ratio (aHR) of incident proteinuria. The age-, sex-, body size-, and history of diabetes and hypertension medication-adjusted percentile rank of baseline eGFR was associated with the aHR of incident proteinuria after adjustment for age, sex, smoking status, regular exercise, regular alcohol consumption, known history of diabetes and/or hypertension medication, body mass index, systolic blood pressure, fasting serum glucose, serum triglycerides, and serum high-density lipoprotein-cholesterol. The relationship between the percentile rank of baseline eGFR and the aHR of incident proteinuria was evaluated with a general additive model. The shaded area represents the 95% confidence interval. Lower panel. Association between RHF (see Methods for details) and aHR of incident proteinuria. The age-, sex-, body size-, and history of diabetes and hypertension medication-adjusted RHF was associated with the aHR of incident proteinuria after adjustment for age, sex, smoking status, regular exercise, regular alcohol consumption, known history of diabetes and/or hypertension medication, body mass index, systolic blood pressure, fasting serum glucose, serum triglycerides, and serum high-density lipoprotein-cholesterol.

**Fig 2:**
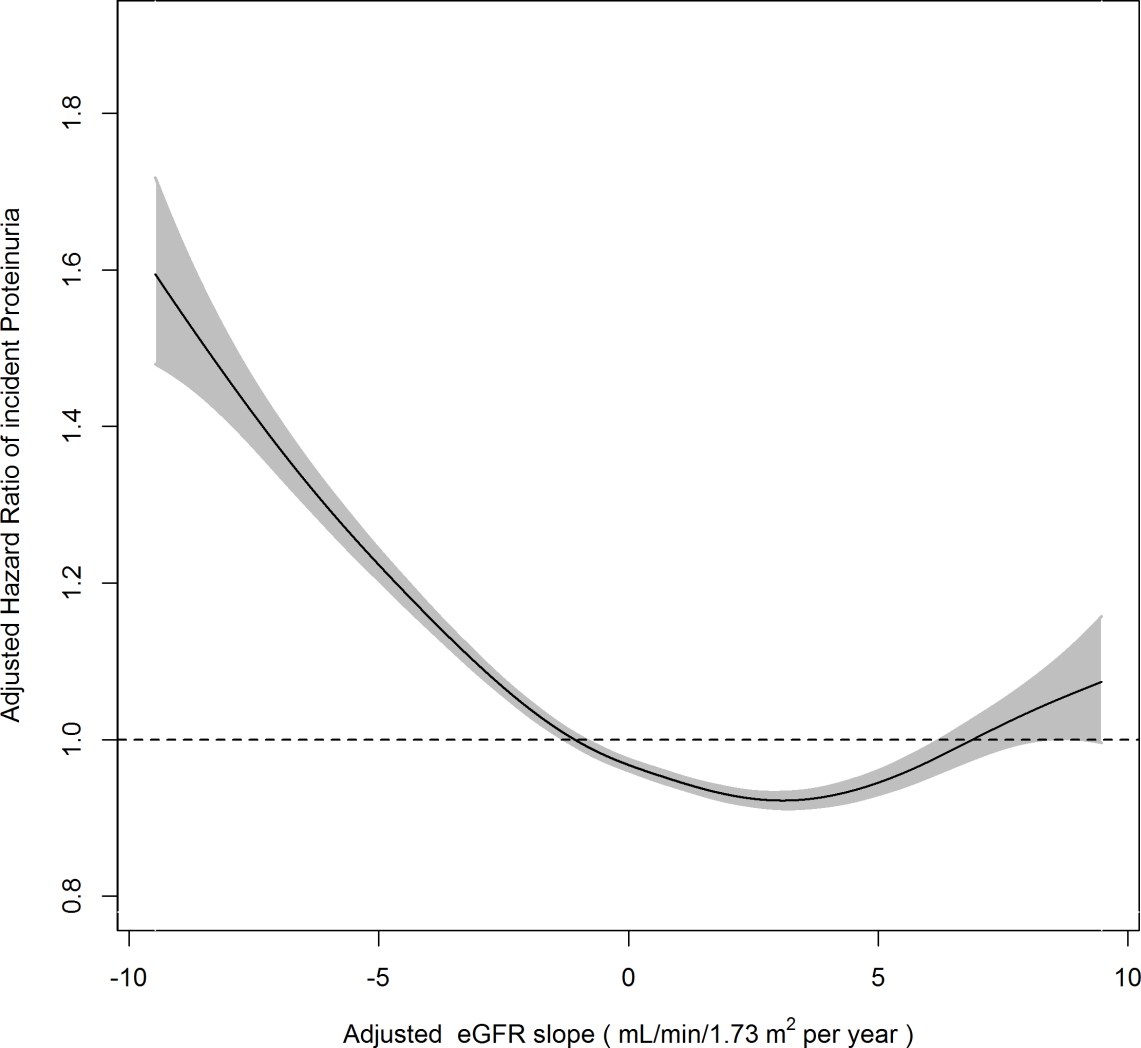
A reverse J-shaped association between estimated glomerular filtration rate (eGFR) slope and the adjusted hazard ratio (aHR) for incident proteinuria. aHR was calculated with a Cox regression model adjusted for age, sex, smoking status, regular exercise, regular alcohol consumption, known history of diabetes and/or hypertension medication, body mass index, systolic blood pressure, fasting serum glucose, serum triglycerides, and serum high-density lipoprotein-cholesterol in subjects who had undergone health screening five or more times. The relationship between the eGFR slope and the aHR was evaluated with a generalized additive model. The shaded area represents the 95% confidence interval.

During 55,346,703 person-years, 426,027 cases of incident proteinuria were observed (Table 2). A reverse J-shaped association between the percentile rank of eGFR residuals and aHR of incident proteinuria was observed, and both the eGFR residuals lower than the 10^th^ percentile and higher than the 80^th^ percentile were associated with higher aHR of incident proteinuria (Fig 1). In the Cox regression model, the aHR for incident proteinuria of RHF was 1.083(95% CI 1.069~1.097) compared to that of non-RHF (Fig 1). With subgroup analysis, although RHF was associated with incident proteinuria irrespective of diabetic status, the risk was lower in non-diabetic than in diabetic subjects (aHR 1.063, 95% CI 1.048~1.079 in non-diabetic subjects; aHR 1.136, 95% CI 1.103~1.169 in diabetic subjects; p for interaction <0.001; Table 2). This association was not observed in women (aHR 1.102, 95% CI 1.084~1.120 in men; aHR 1.005, 95% CI 0.983~1.027 in women; p for interaction <0.001; Table 2 and Fig 1). The difference between age groups was not observed in this association (p for interaction = 0.411; Table 2). When the subjects with a history of hypertensive or diabetic medications were excluded or when the incident proteinuria was defined as 2+ or higher in urine dipstick test for proteinuria, nearly identical results were observed (aHR with no history of hypertensive or diabetic medications; 1.098, 95% CI 1.082 ~ 1.115 and aHR with the incident proteinuria defined as 2+ or higher; 1.086, 95% CI 1.059 ~ 1.113). When the incident proteinuria was defined as 1+ or higher in urine dipstick test for proteinuria at two consecutive tests, the aHR for incident proteinuria was 1.127 (95% CI 1.052~1.206) in subjects who had undergone health screening five or more times. A reverse J-shaped relationship between the eGFR slope and aHR of incident proteinuria was observed and the lowest aHR for the incident proteinuria was observed between 0 and 5 mL/min/1.73 m^2^ per year (Fig 2).

**Table 2:** The association of renal hyperfiltration (RHF)^a^ with higher hazard ratios for incident proteinuria.

		Incident proteinuria					
		Case/	Incidence density^b^^)^	Hazard ratio^c^^)^ (95% CI^d^^)^)			P for interaction
		Person-Year		Model 1^e^^)^	Model 2^f^^)^	Model 3^g^^)^	
Total		426,027/	769.7	1.153	1.090	1.083	-
		55,346,703		(1.138~1.168)	(1.076~1.104)	(1.069~1.097)	
Subgroup by sex	Men	247,515/	816.0	1.202	1.113	1.102	<0.001
		30,331,754		(1.183~1.222)	(1.095~1.131)	(1.084~1.120)	
	Women	178,512/	713.6	1.051	1.005	1.005	
		25,014,949		(1.028~1.074)	(0.983~1.027)	(0.983~1.027)	
Subgroup by median age^h^^)^	Young	193,984/	722.6	1.185	1.091	1.088	0.411
		26,846,303		(1.162~1.231)	(1.170~1.112)	(1.067~1.109)	
	Old	232,044/	814.2	1.126	1.072	1.065	
		28,500,400		(1.106~1.145)	(1.054~1.091)	(1.046 ~1.083)	
Subgroup by diabetic status^i^^)^	Diabetic	66,544/	1659.9	1.196	1.138	1.136	<0.001
		4,008,869		(1.162~1.231)	(1.105~1.171)	(1.103~1.169)	
	Non-diabetic	359,483/	700.2	1.106	1.069	1.063	
		51,337,833		(1.090~1.122)	(1.053~1.085)	(1.048~1.079)	

^a^ see Methods for details.

^b^ per 100,000 person-years.

^c^ Adjusted hazard ratio of RHF for incident proteinuria, compared to non-RHF, by Cox regression model.

^d^ confidence interval.

^e^ Model 1, adjusted for age and sex.

^f^ Model 2, adjusted for age, sex, body mass index, known history of medication for diabetes and/or hypertension, and fasting serum glucose.

^g^ Model 3, adjusted for age, sex, body mass index, known history of medication for diabetes and/or hypertension, smoking status, regular alcohol consumption, regular exercise, systolic blood pressure, fasting serum glucose, serum triglycerides, and serum high-density lipoprotein-cholesterol.

^h^ 44 years in men, 49 years in women.

^i^ diabetics, fasting serum glucose 126 mg/dL or above and/or anti-diabetic medication.

## Discussion

This study of nationwide health screening data comprising about one-third of the whole adult population in South Korea observed that RHF was associated with higher aHR of incident proteinuria, after adjusting for age, sex, history of hypertensive or glucose-lowering medications, regular exercise, regular alcohol consumption, BMI, systolic blood pressure, fasting serum glucose, serum triglycerides, and high-density lipoprotein-cholesterol. The association between RHF and incident proteinuria was observed not only in diabetic but also in non-diabetic subjects. The risk was lower in non-diabetic than in diabetic subjects. The association was not observed in women.

The association between proteinuria and mortality is well established [28, 29]. The natural course of diabetic nephropathy, in which the initial phase of RHF is commonly followed by a progressive increase in urinary albumin excretion and finally by progressive decrease in GFR [30], is also well established although decreased GFR in the absence of albuminuria in diabetic nephropathy has recently been reported [31]. The relationship between RHF and proteinuria or albuminuria has not yet been clearly elucidated in conditions other than diabetes [29]. This study observed an association between RHF and incident proteinuria even in non-diabetic subjects, and to the best of our knowledge, this is the first report on the association between RHF and incident proteinuria in the general population. The explanation of the association between RHF and incident proteinuria is not yet clear. In animal studies, several mechanisms including enlarged radius of the glomerular pores, endothelial dysfunction due to the wall stress, and podocyte damage due to glomerular hypertension have been proposed [32, 33]. RHF associated with excess body weight and/or central obesity has been explained with several mechanisms, including activation of the renin-angiotensin-aldosterone system [34]. It is well known that blockade of the renin-angiotensin-aldosterone system has a reno-protective effect beyond its effect on blood pressure in proteinuric CKD [35]. Therefore, shared pathogenetic mechanisms such as the activation of renin-angiotensin-aldosterone system may explain the association between RHF and incident proteinuria. The association between RHF and incident proteinuria was only observed in men, and there is no available explanation for this differential effect according to sex. Many conflicting reports on gender difference in the prevalence and general tendency of more rapid progression of renal dysfunction in men than in women have attempted to explain this gender effect by differences in dietary factors, kidney size, glomerular hemodynamics, and sex hormones [36]. The possible gender difference in the association between RHF and incident proteinuria observed in this study requires further confirmation.

This study found that both the rapid increase and decline of eGFR were associated with higher aHR of incident proteinuria. Turin et al. reported that the rapid increase of eGFR was associated with higher mortality over 2.5 years of median follow-up compared to stable eGFR, and they explained this association with muscle wasting due to chronic debilitating conditions [37]. Another recent study reported an association of longitudinal increase in measure GFR (using iohexol clearance) with incident albuminuria in non-diabetics [38] is consistent with our observation. These results suggest that the development or progression of RHF and the activation of some shared pathophysiological mechanism(s) between RHF and proteinuria, other than over-estimation of true GFR due to muscle wasting, may be another possible explanation for the association between increasing eGFR and higher mortality.

The massive size of this study population of more than eleven million adults, which covered approximately one-third of the whole adult population in Korea, enabled us to observe renal outcomes in a relatively short follow-up period and to perform subgroup analyses according to sex and age as well as various sensitivity analyses. Despite this strength, this study has several limitations. First, proteinuria was diagnosed with dipstick test not with urine albumin-to-creatinine ratio. Although urine albumin-to-creatinine ratio is a preferred method to detect proteinuria, the urine dipstick test have shown comparable results in many cohort studies on the association between renal measures and cardiovascular outcomes [39]. Second, true GFR was not measured. It was not practical to measure true GFR in this large population. Third, although the population of this study covered one-third of the whole adult population of Korea, the subjects with poor health condition might not participate in health screenings and may therefore be underrepresented in the population of this study. This study excluded the subjects with baseline eGFR less than 60 mL/min per 1.73 m^2^ and/or baseline proteinuria and the sensitivity analysis with further exclusion of the subjects with a history of medication due to hypertension and/or diabetes did not change the results. Therefore, the bias caused by the underrepresentation of the subjects with poor health condition might not influence the association between RHF and the change in eGFR and incident proteinuria. Finally, the observations of this study were from a single ethnicity, and the generalization of these observations should be undertaken with caution.

In conclusion, this study on a population of more than eleven million relatively healthy adults observed that RHF was associated with incident proteinuria not only in diabetic but also in non-diabetic subjects, but not in women, and that increasing GFR as well as decreasing GFR was associated with incident proteinuria. Prospective studies are needed to determine whether the measures to alleviate RHF can prevent the development of CKD.
